# Urine NGAL and KIM-1—Tubular Injury Biomarkers in Long-Term Survivors of Childhood Solid Tumors: A Cross-Sectional Study

**DOI:** 10.3390/jcm10030399

**Published:** 2021-01-21

**Authors:** Eryk Latoch, Katarzyna Konończuk, Katarzyna Muszyńska-Rosłan, Katarzyna Taranta-Janusz, Anna Wasilewska, Edyta Szymczak, Justyna Trochim, Maryna Krawczuk-Rybak

**Affiliations:** 1 Department of Pediatric Oncology and Hematology, Medical University of Bialystok, 15-274 Białystok, Poland; kononczukk@gmail.com (K.K.); kmroslan@post.pl (K.M.-R.); rybak@umb.edu.pl (M.K.-R.); 2 Department of Pediatrics and Nephrology, Medical University of Bialystok, 15-274 Białystok, Poland; katarzyna.taranta@wp.pl (K.T.-J.); annwasil@interia.pl (A.W.); edytaszymczak91@gmail.com (E.S.); 3 Department of Pediatric Laboratory Diagnostics, Medical University of Bialystok, 15-274 Białystok, Poland; justynatrochim@wp.pl

**Keywords:** urine KIM-1, urine NGAL, childhood solid tumors, nephrotoxicity, chronic kidney diseases, CKD, childhood cancer survivors, CCS

## Abstract

The deterioration of renal function after childhood solid tumors treatment is the result of using the intensive multimodal therapy. In recent years, urinary kidney injury molecule-1 (KIM-1) and urinary neutrophil gelatinase-associated lipocalin (NGAL) have been introduced as potential promising biomarkers of early kidney damage. The aim of the present study was to determine whether anticancer treatment has any effect on the concentration of KIM-1 and NGAL and its association with renal impairment in survivors of childhood solid tumors. Sixty patients previously treated for solid tumors were involved in this study. The median time after end of treatment was 8.35 years. Urine KIM-1 and NGAL levels were measured using immunoenzymatic ELISA commercial kits. Higher levels of urine NGAL, KIM-1/cr. (creatinine), and NGAL/cr. ratios were found in comparison with healthy controls (*p* < 0.0001). Among all subjects, 23% were found to have decreased estimated glomerular filtration rate (eGFR). A strong correlation between KIM-1/cr. and a cumulative dose of ifosfamide was observed (r = 0.865, *p* < 0.05). In addition, a moderate correlation between NGAL/cr. and a cumulative dose of cisplatin was identified (r = 0.534, *p* < 0.05). The AUC for KIM-1/cr. was 0.52, whereas NGAL/cr. showed a diagnostic profile describing the AUC of 0.67. Univariable regression showed significant associations between NGAL/cr. ratio and subjects after unilateral nephrectomy (coeff. 63.8, *p* = 0.007), cumulative dose of cisplatin (coeff. 0.111, *p* = 0.033), and age at diagnosis (coeff. 3.75, *p* = 0.023). The multivariable model demonstrated only cumulative dose of cisplatin as an independent factor influence on NGAL/cr. ratio. The results of our study showed increased levels of urine KIM-1 and NGAL many years after completion of the childhood solid tumors treatment, which correlated positively with a cumulative dose of ifosfamide and cisplatin. This study also suggests that unilateral nephrectomy could affect the concentration of the studied biomarkers.

## 1. Introduction

In recent decades, the number of childhood cancer survivors (CCS) has increased significantly with the improvement of diagnostic and treatment methods with the cure rate of over 80% for all types of cancers. However, these patients experience many health problems after the end of the treatment. Any organ or system may be affected due to anticancer treatment and cancer itself [1,2,3]. Renal adverse effects are one of the late sequelae with prevalence ranging from 0 to 84% depending on the study population [4].

Nephrotoxicity is the result of using the multimodal therapy, including cytostatics (i.e., cyclophosphamide, ifosfamide, cisplatin, methotrexate), abdominal radiotherapy, immunotherapy, surgery (nephrectomy) and supportive treatment, which may adversely affect the kidneys (amphotericin, aminoglycosides, furosemide). The vast majority of these drugs are administered during childhood solid tumors (CST) treatment. Thus, survivors of CSTs are particularly vulnerable to the development of impaired renal function later in life. Kidney injury may have many distinct clinical pictures, including reduced estimated glomerular filtration rate (eGFR), loss of tubular function, and hypertension [5,6,7]. In the beginning, a decline in renal function may be clinically silent and may even progress toward the end-stage kidney diseases over time. Moreover, commonly used methods of evaluating kidney function do not allow for early detection of renal deterioration. Serum creatinine is ubiquitously used to estimate the glomerular filtration rate. However, its concentration can be affected by lean body mass, age, sex, ethnicity, hydration status, and dietary protein intake [8,9]. On the other hand, there are no suitable, widely used markers of tubular damage. Due to all these limitations, new biomarkers to assess tubular function of the kidney are recently being sought. The introduction of new diagnostic tools can be beneficial, especially for individuals who have been treated with nephrotoxic drugs, for instance, due to anticancer treatment. The ideal biomarker should be non-invasive with high sensitivity and specificity to detect early kidney damage. In recent years, urinary kidney injury molecule-1 (KIM-1) and urinary neutrophil gelatinase-associated lipocalin (NGAL) have been introduced as potential promising biomarkers with high prognostic profile [10].

KIM-1 is a type of I cell transmembrane glycoprotein, which is produced in the kidneys and is not detectable in healthy subjects. It was observed that renal ischemic or toxic injury increased the level of KIM-1 by the up-regulation of mRNA in proximal tubule cells. Most studies investigated the role of KIM-1 as a biomarker for various etiologies of acute kidney injury (AKI), including cisplatin-induced injury, showed KIM-1 to be a promising predictor [11].

NGAL is a member of the lipocalin family that is secreted by a different type of human tissue, including kidneys, gastrointestinal tract, respiratory system, and neutrophils. In kidneys, NGAL is excreted from the loop of Henle epithelial cells and, in healthy people, it is detectable in urine at a very low level. Similar to KIM-1, the increase in the excretion of urine NGAL occurs in response to the renal tubular damage (ischemic or toxic) and depends on its degree. Various studies have highlighted that urinary NGAL is a sensitive biomarker of renal damage in AKI patients as well as other kidney diseases such as tubulointerstitial nephritis, diabetic nephropathy, or nephrotic syndrome [12].

There is a limited number of published studies assessing the applicability of KIM-1 and NGAL as early biomarkers of kidney injury in long-term childhood solid tumor survivors with a subtle deterioration of renal function undetectable by standard methods. From an individual and social point of view, rapid diagnosis and prompt implementation of the treatment are vital to preserve normal kidney function and to maintain the quality of life and lifespan of CST survivors. 

The aim of the present study was to determine whether anticancer treatment used in childhood has any effect on the concentration of urinary kidney injury molecule-1 (KIM-1) and neutrophil gelatinase-associated lipocalin (NGAL) in survivors of childhood solid tumors many years after treatment.

## 2. Materials and Methods

**Figure 1:**
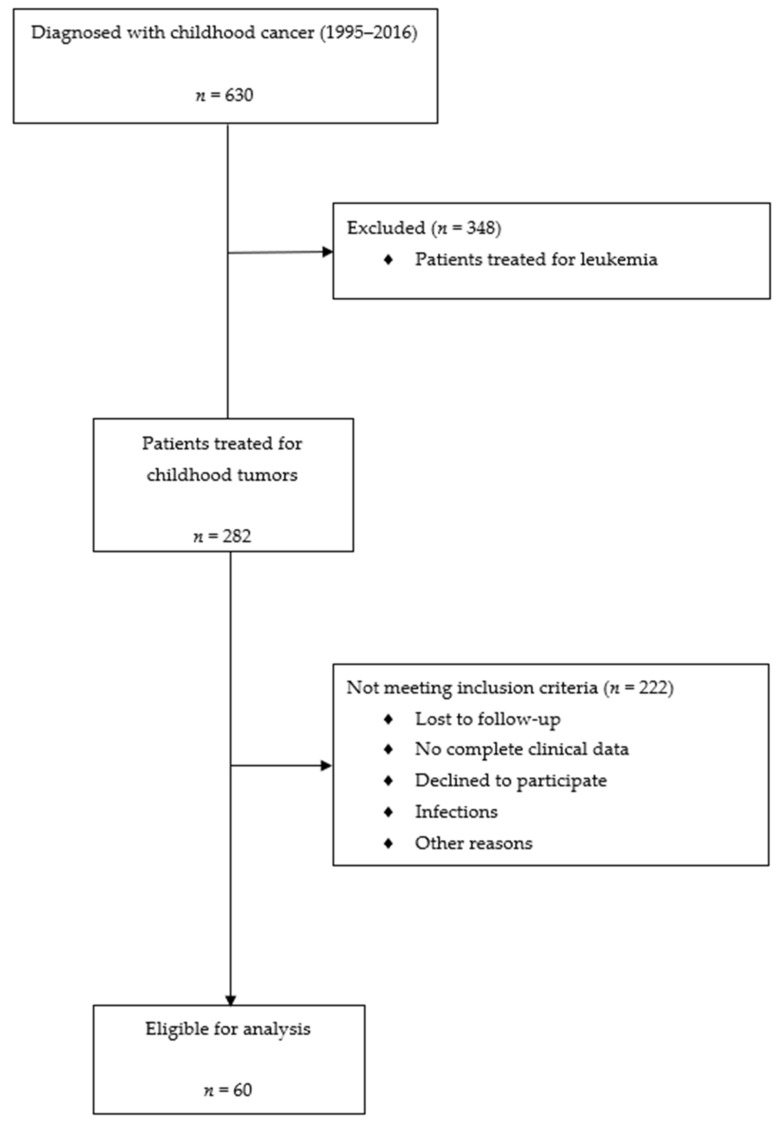
Flow chart describing the design of the cross-sectional study conducted among survivors of childhood solid tumors.

Sixty subjects (27 males, 33 females) visiting the follow-up outpatients’ clinic for childhood cancer survivors at the Department of Pediatric Oncology and Hematology, Medical University of Bialystok (Poland), from 2018 to 2019, were involved in this study. They were treated between 1995 and 2016 in childhood for various types of solid tumors (Figure 1). Inclusion criteria were the following: (1) diagnosed with cancer under the age of 18 years, (2) complete remission, (3) availability of complete clinical data. Subjects with congenital anomalies of kidney or urinary tract, current infection, or relapse of cancer were excluded from the study. All the children were treated according to the international protocols approved by the Polish Pediatric Solid Tumors Group. Written informed consent was obtained from the participants or their parents/guardians. The study was approved by the Institutional Review Board of the Medical University of Bialystok in accordance with the Declaration of Helsinki (R-I-002/62/2018).

For all participants, careful clinical history, including demographic information, comorbidities, data associated with treatment, such as used nephrotoxic cytostatics (cyclophosphamide, ifosfamide, cisplatin, methotrexate), and abdominal radiotherapy, was derived from the CCS database. All patients underwent a full physical examination and had a normal ultrasound of the kidney and urinary tract performed by an experienced radiologist. Anthropometric traits were collected using standard procedures. Body mass index (BMI) was calculated using the following formula: weight (kg) / height^2^ (m^2^). Blood pressure (systolic (SBP) and diastolic (DBP)) were measured three times using a standardized sphygmomanometer with 2 min intervals. Hypertension was defined as a mean SBP and/or DBP level equal or above 95th percentile adjusted for age, sex, and height. The control group consisted of 53 age-matched healthy peers (24 males, 29 females) who were offspring of the department’s employees and healthy school pupils recruited form the OLAF study [13]. This group of healthy individuals has already been used in our previous research on biomarkers of kidney damage carried out in a cohort of acute lymphoblastic leukemia patients [14].

After a 12 h night, fasting peripheral blood was collected from each participant for routine laboratory testing. Clean catch urine samples were stored at −80 °C for further analysis. The serum creatinine level was measured by enzymatic method, and the estimated glomerular filtration rate (mL/min/1.73m^2^) was calculated using the updated Schwartz formula: eGFR = 0.413 x (height in cm / serum cr. in mg/dL). Urine KIM-1 and NGAL levels were measured using commercial immunoassays (R&D SYSTEMS a bio-techne brand, Quantikine^®^ ELISA, Minneapolis, USA) according to the instructions for the ELISA kit. The tested urine biomarkers were calculated per milligram urine creatinine (cr.) in order to avoid the effect of urine dilution (KIM-1/cr. and NGAL/cr. ratios, respectively). Urine albumin concentration was determined by Lowry’s method. Subjects with a urinary albumin/creatinine ratio between 30 and 300 μg/mg were considered to have albuminuria. The stages of chronic kidney disease (CKD) were classified according to Kidney Disease: Improving Global Outcomes (KDIGO) guidelines, and it is defined by structural or functional abnormalities of the kidney for ≥3 months, with or without decreased eGFR or eGFR <60 mL/min/1.73m^2^ for ≥3 months, with or without kidney damage [15].

All continuous variables were tested for normal distribution using Shapiro–Wilk tests. Data were expressed as mean and standard deviation (SD) or median (Me) and interquartile range (IQR) when appropriate. In the analysis of the categorical variables, chi-square test or Fisher exact test was used. Continuous variables were compared with the Student *t*-test or Mann–Whitney *U*-test depending on the normal distribution. Univariable and multivariable linear regression models were used to examine the association between dependent variables (KIM-1, KIM-1/Cr., NGAL, NGAL/Cr.) and independent variables postulated as having a potentially negative impact on kidney function. The correlations between KIM-1/cr. and NGAL/cr. and a cumulative dose of cytostatics were calculated by Spearman correlation coefficients. The receiver operating characteristic (ROC) curve was used to establish the diagnostic value of the urine biomarkers and the optimum cut-off values. Statistical analysis was performed using STATA 12.1 version (StatCorp, College Station, Texas, USA), and statistical significance was determined at 0.05. 

## 3. Results

The demographic characteristics of the study group are presented in Table 1. The median time at the start of the treatment was 15.5 years (range, 4 months–17.9 years) and the median follow-up time from the end of the treatment was 8.35 years (range, 3 months–24 years). There were no statistical differences between the study group and the reference group. All participants had normal eGFR prior to treatment.

**Figure 2:**
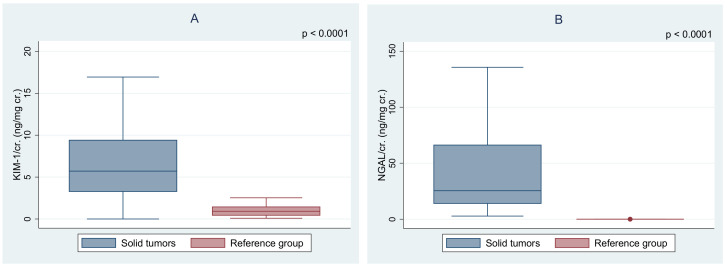
Comparison of (**A**) the urine KIM-1/cr. ratio (kidney injury molecule-1/creatinine) and (**B**) the urine NGAL/cr. ratio (neutrophil gelatinase-associated lipocalin/creatinine) between the solid tumor survivors and the reference group.

In the group of solid tumors, significantly higher levels of urine NGAL, KIM-1/cr., and NGAL/cr. ratios were found in comparison with healthy peers (Figure 2, Table 2). A summary of the biochemical parameters of the study group according to sex is presented in Table 3. Females had a higher level of NGAL and NGAL/cr. ratio than male subjects (*p* < 0.001). The age at diagnosis and time elapsed since the end of the treatment were not correlated with the level of KIM-1 and NGAL. Among all subjects, 14 (23%) were found to have decreased eGFR: 13 below 90 mL/min/1.73m^2^ and 1 between 30 and 59 mL/min/1.73m^2^. Additionally, we retrospectively analyzed data about eGFR measurement at least 3 months before the study time. All subjects with eGFR <90 mL/min/1.73m^2^ at the time of the study also had decreased eGFR at least three months earlier. One of all participants with eGFR <60 mL/min/1.73m^2^ met the criteria of CKD according to KDIGO guidelines.

No differences in the level of urine biochemical parameters between the subset of participants with decreased eGFR (< 90 mL/min/1.73m^2^) and those who had normal eGFR (> 90 mL/min/1.73m^2^) were observed (Table 4). Univariable linear regression analysis did not show any impact of eGFR on NGAL, NGAL/cr., KIM-1, and KIM-1/cr. Nine subjects (15%) were hypertensive. Moreover, no relationship was identified between albuminuria and urine biomarkers as well as between hypertensive patients and KIM and NGAL level and their ratios.

Taking into account the fact that the results may differ in patients after kidney removal, we conducted a separate analysis in CCS with solitary functioning kidney. In this subgroup, we did not show any significant changes in the level of urine biomarkers or fasting serum creatinine, as shown in Table 5. However, univariable linear regression analysis showed significant correlations between NGAL/cr. and individuals after simple nephrectomy (coeff. 63.8, *p* = 0.007, 95% CI 18.61–109.02), cumulative dose of cisplatin (coeff. 0.111, *p* = 0.033, 95% CI 0.01–0.21), and age at study (coeff. 3.75, *p* = 0.023, 95% CI 0.53–6.96). Other independent variables such as age at diagnosis (coeff. 1.67, *p* = 0.370, 95% CI −2.02–5.36), follow-up time (coeff. 2.46, *p* = 0.184, 95% CI −1.2–6.14), cumulative dose of cytostatic agents—cyclophosphamide (coeff. −0.02, *p* = 0.06, 95% CI −0.04–0.01), ifosfamide (coeff. 0.001, *p* = 0.4, 95% CI −0.001–0.001), methotrexate (coeff. 0.001, *p* = 0.321, 95% CI −0.01–0.01), and abdominal radiotherapy (coeff. 22.91, *p* = 0.298, 95% CI −20.73–66.56) did not affect the NGAL/cr. ratio. Confounding factors in the univariable analyses at *p* ≤ 0.05 were included in multivariable model (Table 6). Similar analyses were conducted for KIM-1/cr. ratio and nephrectomy (coeff. −0.017, *p* = 0.994, 95% CI −4.71–4.66), age at study (coeff. −0.320, *p* = 0.06, 95% CI −0.63–0.13), age at diagnosis (coeff. −0.078, *p* = 0.662, 95% CI −0.43–0.28), time after end of treatment (coeff. −0.34, *p* = 0.055, 95% CI −0.69–0.01), cumulative dose of cyclophosphamide (coeff. −0.001, *p* = 0.597, 95% CI −0.01–0.01), ifosfamide (coeff. 0.0001, *p* = 0.001, 95% CI 0.0001–0.002), cisplatin (coeff. 0.002, *p* = 0.719, 95% CI −0.01–0.01), methotrexate (coeff. 0.001, *p* = 0.347, 95% CI −0.01–0.01), and abdominal radiotherapy (coeff. 1.14, *p* = 0.591, 95% CI −3.08–5.37). Due to the fact that in univariable analysis, potential confounding factors did not affect significantly the KIM-1/cr. ratio, the multivariable model was not built.

ROC analyses were performed to determine the diagnostic profile of both ratios, KIM-1/cr. and NGAL/cr., in identifying CCS with reduced renal function —eGFR < 90 mL/min/1.73m^2^. The AUC for the KIM-1/cr. ratio was 0.52 (95% CI: 0.33–0.69) with the best cutoff value of 1.74 ng/mL (sensitivity 100%, specificity 18.92%), while NGAL/cr. showed a diagnostic profile describing the AUC of 0.67 (95% CI: 0.51–0.83) with the best cutoff value of 5.17 ng/mL (sensitivity 100%, specificity 8.11%).

Due to the fact that some cytostatic agents such as cyclophosphamide, ifosfamide, cisplatin, or methotrexate may induce deterioration of kidney function, we performed the analysis investigating the association between the type of used cytostatics and the number of patients with decreased eGFR (< 90 mL/min/1.73m^2^). Nevertheless, we did not show any significant differences.

**Figure 3:**
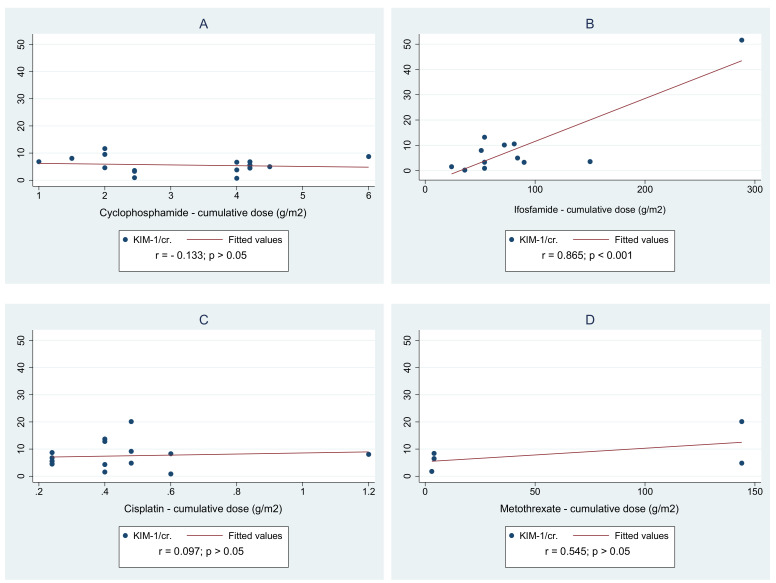

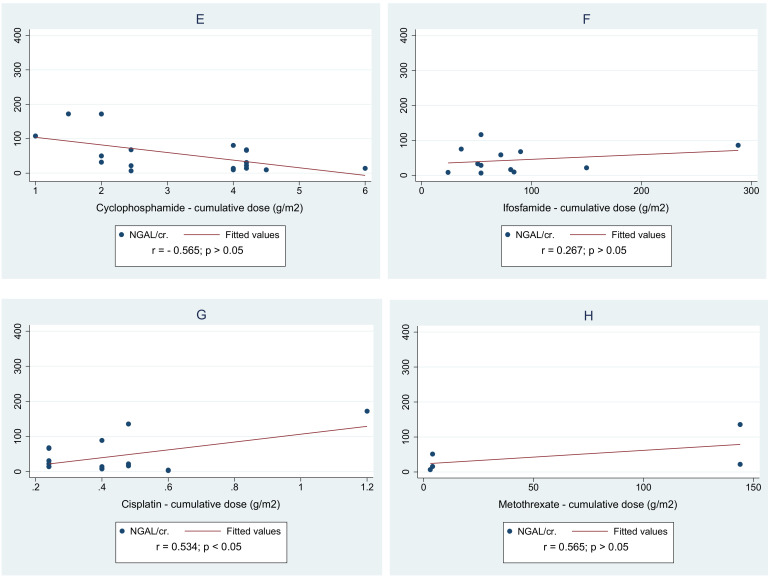
Spearman correlations of urine biomarkers ratios: KIM-1/cr. (**A**–**D**) and NGAL/cr.(**E**–**H**) and cumulative dose of cytostatics used during chemotherapy in childhood.

Further analysis checked the correlations of urine biomarkers ratios (KIM-1/cr. and NGAL/cr.), and a cumulative dose of cytostatics used during chemotherapy in CCS was performed (Figure 3). A strong correlation between KIM-1/cr. ratio and a cumulative dose of ifosfamide was found (r = 0.865, *p* < 0.05). In addition, a moderate correlation between NGAL/cr. ratio and a cumulative dose of cisplatin was observed (r = 0.534, *p* < 0.05). The mean level of urine biomarkers (KIM-1, NGAL) and its ratios (KIM-1/cr., NGAL/cr.) did not differ among the study participants exposed to abdominal radiotherapy during childhood from those who were treated with chemotherapy only. We did not carry out the analysis between the types of cancers, as these groups were underrepresented for meaningful evaluations. Moreover, due to the fact that children in the first years of life may be more exposed to drug-induced nephrotoxicity, the study group was divided according to the time of diagnosis (0–3 years vs. 3 years and more); however, no differences in the concentration of the KIM-1 and NGAL were found. The independent factors that were found to have a significant correlation with urine NGAL/cr. and KIM-1/cr. in univariable regression were used as explanatory variables to create a multiple regression model. It resulted in the creation of the model presented in Table 6. Of all the factors included in the analysis, only a cumulative dose of cisplatin significantly impacts on the NGAL/cr. ratio.

## 4. Discussion

In the last two decades, many markers have been tested to find the most sensitive and specific indicators of drug-induced nephrotoxicity [5,6,16]. These studies resulted in the approval of selected candidates for biomarkers for use in a preclinical application for regulatory decision making [10]. On the basis of the fact that there is an urgent need for improved diagnostic tools to predict nephrotoxicity in CCS in this study, we focused on the best promising urinary proteins (KIM-1 and NGAL) based on previous studies. Most available data conducted so far have focused exclusively on AKI. Data from the literature indicate that the elevated urine excretion of KIM-1 and NGAL was observed directly after the administration of cisplatin or ifosfamide and was proportional to the severity of tubular damage [17,18]. Another two-center pilot study (Canada, USA) by Sterling et al. prospectively assessing the utility of the NGAL, serum creatinine, and interleukin-18 (Il-18) levels in children receiving chemotherapy (cisplatin/carboplatin or ifosfamide) showed that NGAL and Il-18 were very good predictors of drug-induced AKI [19]. Li et al. at research assessing the value of serum NGAL in identifying early acute kidney injury induced by a high dose of methotrexate (HDMTX) concluded that the combination of serum creatinine ratio and serum NGAL after 24 h of cytostatic infusion had higher value for early diagnosis of HDMTX associated with AKI compared with serum creatinine only [20].

The role of the urinary biomarkers in identifying early stages of kidney injury is still unclear [11,21,22,23,24]. Data from the literature indicate a significant relationship between a decline in renal function of different etiologies and increased NGAL level. These conditions included acute lymphoblastic leukemia (ALL), CKD, glomerulonephritis, autosomal dominant polycystic kidney disease (ADPKD), IgA nephropathy, and pediatric systemic lupus erythematosus. Moreover, they suggest that NGAL (serum and urine) may be a good predictor of CKD progression to end-stage kidney disease [14,21,25,26]. Similar observations were reported in research assessing the usefulness of KIM-1 and NGAL in children with congenital hydronephrosis. The authors suggested that the increased urine KIM-1 and NGAL levels were associated with worsening obstruction [27]. In contrast, in the study by Seiber et al. carried out on adult patients with CKD caused by diabetes, ADPKD, hypertension, or glomerulonephritis, the authors did not confirm the predominance of NGAL and KIM-1 over albuminuria [11].

In this prospective cohort study carried out on childhood solid tumor survivors, 23% of subjects with decreased eGFR (<90 mL/min/1.73m^2^) were found many years after the end of the treatment. Our results are consistent with some, but not all, previous reports, and they provide further evidence that treatment-related factors impair renal function in CST survivors [27,28]. On the other hand, one of the available large-scale studies, performed on 1122 participants, showed reduced eGFR and higher glomerular dysfunction probability up to 35 years in 6.6% of subjects after cancer diagnosis compared with CCS treated without nephrotoxic cytostatics [29]. This discrepancy may be due to the fact that our study involved only patients treated for solid tumors, who are more exposed to the nephrotoxic treatment than children treated for other more often cancers, i.e., acute lymphoblastic or myeloid leukemia.

To our best knowledge, this is the first clinical study of the association of tubular injury biomarkers (urine KIM-1 and NGAL) in survivors of childhood solid tumors many years after the end of the treatment. In this study, a significantly increased urinary excretion of NGAL as well as higher NGAL/cr. and KIM-1/cr. ratio in CST survivors were found, as compared to the reference group. This finding may indicate renal tubular damage many years after anticancer treatment. We also noticed that females have a significantly higher level of NGAL and NGAL/cr. ratio than males, which is in accordance with some previous studies. Contrary to expectations, no association between urine biomarkers and eGFR was observed. In the study conducted in adults (mean age 57 ± 16), Bolignano et al. demonstrated that NGAL closely reflects the entity of renal impairment in patients with CKD and represents a strong risk marker for progression of CKD [30]. It is noteworthy that in a subset of participants with reduced eGFR, nearly all of them (except one) had mildly decreased eGFR in the range from 60 to 90 mL/min/1.73m^2^. This may also be due to the fact that different kidney cells have different susceptibility to damage depending on the treatment applied. Certain chemotherapeutics used in the treatment of childhood solid tumors may primarily damage tubule cells and then progressively lead to the deterioration of glomerular function. Moreover, particular cytostatic agents, such as ifosfamide in high doses, might damage tubular function already during the intensive treatment, which may result in the development of CKD later in life. Hence, it is likely that these individuals may suffer first from tubular damage, which is not demonstrated by routine eGFR measurement.

In addition, we performed a separate analysis for those survivors who underwent a unilateral nephrectomy. Univariable regression analysis showed significant correlation between NGAL/cr. and individuals after simple nephrectomy, but the size of the subset of subjects was relatively small. Due to lack of data, further research is needed in this area. Likewise, older age at study correlated positively with a higher level of NGAL/cr.; however, the time elapsed since the cessation of treatment seems to be too short to draw a conclusion. Moreover, we also performed single regression and correlation analyses of KIM-1/cr. and NGAL/cr. with a cumulative dose of cytostatics used during the therapy. In CST survivors, we found a positive correlation between KIM-1/cr. ratio and a cumulative dose of ifosfamide as well as NGAL/cr. ratio and a cumulative dose of cisplatin used during childhood. These results suggest that the two most commonly used chemotherapeutics in the treatment of solid tumors (ifosfamide, cisplatin) may affect the deterioration of kidney function. Data from the literature confirm tubular damage immediately or a few days after the infusion of cisplatin or ifosfamide; however, there are no data on long-term survivors of childhood solid tumors [10,17,18]. Recent attention has been focused on findings that anticancer treatment may induce premature senescence of different human tissues due to the intracellular changes, which lead to the earlier development of chronic diseases such as atherosclerosis, osteoporosis, or CKD. [31,32].

There are several limitations in this study. The single-center research should be interpreted in a context of possible bias that may occur. Secondly, due to the fact that the study group consisted of survivors of heterogenous solid tumors that were treated using distinct treatment modalities, it was difficult to determine the impact of each treatment protocol. Therefore, the authors focused mainly on the analysis of the effect of particular cytostatic agents rather than the specific types of tumors. Thirdly, in the current study the same reference group was used as in our previous study for acute leukemia survivors [14]. Finally, the size of the study group was relatively small, which may influence the final results. Another limitation of our study is that GFR was only estimated from creatinine values, instead of being measured by standard techniques, e.g., iohexol plasma clearance. However, these are rapid, accurate, and precise measures of kidney function, which is essential for the daily management of children. The strengths of our research include a relatively long follow-up time and no ethnic diversity.

In summary, the results of our pilot cross-sectional study carried out in survivors of solid tumors showed increased levels of urine KIM-1 and NGAL many years after completion of the treatment, which correlated positively with a cumulative dose of ifosfamide and cisplatin. This study also suggests that unilateral nephrectomy could affect the concentration of the biomarkers studied. However, conclusions from this study should be drawn with caution, as nephrotoxicity remains under several multifactorial environmental effects. Moreover, the utility of KIM-1 and NGAL as early markers of tubular damage many years after childhood solid tumors treatment and influence of cytostatic agents on the kidneys needs to be confirmed in longitudinal large prospective cohort studies.

## Figures and Tables

**Table 1:** Clinical characteristics of the survivors of childhood solid tumors and reference group.

	Study Group	Reference Group
Patients	60 (100%)	53 (100%)
Male	27 (45%)	24 (45%)
Female	33 (55%)	29 (55%)
Age at diagnosis (years)	4.61 (4.95–12.55)	
Age at study (years)	15.5 (9.25–19.00) ^a^	11.5 (8.04–16.5) ^a^
Follow-up after treatment (years)	8.35 (4.95–12.55)	
Diagnosis type		
Wilms tumor	17 (28%)	
Sarcoma	14 (23%)	
Hodgkin lymphoma	10 (17%)	
Neuroblastoma	9 (15%)	
Hepatoblastoma	4 (7%)	
Germ tumors	3 (5%)	
Langerhans cell histiocytosis	3 (5%)	
Chemotherapy		
Cyclophosphamide (cumulative dose (g/m^2^), *n = 19*	4 (2.00–4.20)	
Ifosfamide (cumulative dose in g/m^2^), *n = 12*	63 (51.70–88.50)	
Cisplatin (cumulative dose in g/m^2^), *n = 16*	0.4 (0.24–0.48)	
Methotrexate (cumulative dose in g/m^2^), *n = 5*	4.04 (3.52–14.40)	
Radiotherapy	25 (42%)	
Abdominal radiotherapy (cumulative dose in Grey), *n = 19*	21 (19.8–21)	
No	35 (58%)	

Data are presented as median and interquartile range; categorical variables are presented as numbers (%). ^a^ *p* > 0.05

**Table 2:** Summary of the biochemical parameters of childhood solid tumors and the reference group.

	Solid Tumor Me (IQR)	Reference Group Me (IQR)	*p*
**Total**	60	53	
Serum creatinine (mg/dL)	0.65 (0.53–0.89)	0.49 (0.40–0.63)	< 0.0001
eGFR (mL/min/1.73m^2^)	109.1 (86.52–135.0)	118.1 (102.9–135.6)	0.105
KIM-1 (ng/mL)	0.79 (0.20–1.14)	0.81 (0.41–1.04)	0.890
KIM-1/cr. (ng/mg cr.)	6.16 (3.29–9.98)	0.93 (0.44–1.48)	< 0.0001
NGAL (ng/mL)	3.98 (1.48–11.45)	0.004 (0.001–0.005)	< 0.0001
NGAL/cr. (ng/mg cr.)	31.37 (14.23–84.67)	0.004 (0.002–0.007)	< 0.0001

eGFR, estimated glomerular filtration rate, KIM-1, urinary kidney injury molecule-1, NGAL, urinary neutrophil gelatinase-associated lipocalin, cr. creatinine. Data are given as the median (Me) with interquartile range (IQR).

**Table 3:** Urine concentration of biochemical parameters in survivors of solid tumors according to gender.

	Male	Female	*p*
**Total**	***n* = 27**	***n* = 33**	
Serum creatinine (mg/dL)	0.77 (0.46–0.96)	0.63 (0.53–0.72)	0.252
GFR (mL/min/1.73m^2^)	117.9 ± 37.03	111.1 ± 26.8	0.459
KIM-1 (ng/mL)	0.63 (0.19–0.97)	0.95 (0.44–1.25)	0.075
KIM-1/cr. (ng/mg cr.)	4.82 (1.84–10.14)	6.48 (3.95–9.88)	0.334
NGAL (ng/mL)	2.28 (0.98–5.27)	8.31 (2.70–16.80)	< 0.001
NGAL/cr. (ng/mg cr.)	21.63 (11.97–33.19)	67.83 (21.99–115.7)	< 0.001

eGFR, estimated glomerular filtration rate; KIM-1, urinary kidney injury molecule-1; NGAL, urinary neutrophil gelatinase-associated lipocalin; cr., Creatinine; *n*, number of subjects. Data are given as the median (Me) with interquartile range (IQR).

**Table 4:** Summary of the biochemical parameters of childhood solid tumors survivors according to estimated glomerular filtration rate (eGFR).

	Decreased eGFR < 90/mL/1.73 m^2^ Me (IQR)	Normal eGFR > 90/mL/1.73 m^2^ Me (IQR)	*p*
	*n* = 14	*n* = 46	
KIM-1 (ng/mL)	0.87 (0.39–1.43)	0.78 (0.20–1.21)	0.588
KIM-1/cr. (ng/mg cr.)	6.06 (3.17–13.47)	6.48 (3.34–9.82)	0.827
NGAL (ng/mL)	3.02 (1.32–6.11)	4.51 (1.55–11.80)	0.454
NGAL/cr. (ng/mg cr.)	21.99 (15.84–62.73)	31.86 (14.21–82.58)	0.466

*eGFR,* estimated glomerular filtration rate; *KIM-1*, urinary kidney injury molecule-1; *NGAL*, urinary neutrophil gelatinase-associated lipocalin; *cr.,* creatinine. Data are given as the median (Me) with interquartile range (IQR) or mean ± standard deviation (SD) when appropriate.

**Table 5:** Urine concentration of biochemical parameters in survivors of solid tumors with solitary functioning kidney and normal kidney.

	Solitary Functioning Kidney	Normal Kidneys	*p*
**Total**	15	45	
Serum creatinine (mg/dL)	0.62 (0.45–0.75)	0.66 (0.56–0.95)	0.137
GFR (mL/min/1.73m^2^)	111.2 (95.54–136.4)	104.6 (80.88–128.5)	0.254
KIM-1 (ng/mL)	0.66 (0.19–1.24)	0.87 (0.19–1.58)	0.684
KIM-1/cr. (ng/mg cr.)	4.63 (2.73–12.31)	6.62 (3.58–9.82)	0.983
NGAL (ng/mL)	4.80 (2.13–12.42)	3.28 (1.19–7.28)	0.284
NGAL/cr. (ng/mg cr.)	45.09 (22.14–112.4)	22.13 (13.86–70.71)	0.151

*eGFR*, estimated glomerular filtration rate; *KIM-1*, urinary kidney injury molecule-1; *NGAL*, urinary neutrophil gelatinase-associated lipocalin; *cr.,* creatinine, *OR*, odds ratio.

**Table 6:** Multiple linear regression analysis of the NGAL/creatinine ratio in childhood solid tumors survivors.

Variables	Coefficient	Standard Error	*p*	95% C	
Cisplatin (cumulative dose in g/m^2^)	0.108	0.048	0.041	0.005	0.211
Age at diagnosis (years)	3.165	1.234	0.182	−1.702	8.033
Nephrectomy (no vs. yes)	50.09	44.64	0.284	−47.18	147.3
